# Predictors of physical violence against children in Rwanda: findings from a National Cross-Sectional Survey

**DOI:** 10.1186/s12889-022-14815-0

**Published:** 2022-12-19

**Authors:** Alypio Nyandwi, Namatovu Fredinah, Vincent Rusanganwa, Cyprien Munyanshongore, Laetitia Nyirazinyoye, Prata Ndola, Jean Damascene Nshimiyimana, Marie-Gloriose Ingabire, Nyirabahinde Anastasie, Natasha Salant, Kamukunzi Mecthilde, Hakomeza Emmanuel, Assumpta Mukabutera

**Affiliations:** 1 School of Public Health, University of Rwanda, Kigali, Rwanda; 2 Umea University, Umea, Sweden; 3 University of California, Berkeley, USA; 4 National Rehabilitation Service, Kigali, Rwanda; 5 International Development Research Centre, Ottawa, Canada; 6 Ministry of Gender and Family Promotion, Kigali, Rwanda; 7 Clinton Health Access Initiative, Boston, USA; 8 World Health Organization, Freetown, Sierra Leone; 9 Rwanda Biomedical Centre, Kigali, Rwanda

**Keywords:** Physical violence, Physical abuse, Violence against children, Child abuse, Rwanda

## Abstract

**Background:**

To address the challenges of limited national data on the prevalence and nature of violence experienced by children, Rwanda conducted, in 2015–2016, the first National Survey on Violence among female and male children and youth aged 13–24 years. To further contribute to these efforts to fill existing data gaps, we used the Rwanda survey data to assess the prevalence and predictors of physical violence (PV) in children aged 13–17.

**Methods:**

A nationally representative sample of 618 male and 492 female children were analysed. Nationally representative weighted descriptive statistics were used to analyse the prevalence of PV self-reported by children, and logistic regression models were applied to investigate its predictors.

**Results:**

Sixty percent of all children, including 36.53% of male and 23.38% of female children, reported having experienced any form of PV in their lifetime. Additionally, 21.81% of male children and 12.73% of female children reported experiences of PV within twelve months before the survey date. Older children (OR: 0.53 [0.40–0.72]), female children (OR: 0.43 [0.31–0.58]), and children not attending school (OR: 0.48 [0.31–0.73]) were less likely to be physically abused. However, sexually active children (OR: 1.66 [1.05–2.63]), children in households from the middle wealth quintile (OR: 1.63 [1.08–2.47]), children living in a larger family (OR: 1.55 [1.07–2.26]), and children who reported not feel close to both biological parents (OR: 2.14 [1.31–3.49]) had increased odds of reporting physical violence.

**Conclusion:**

Higher rates of PV in children attending school were the key finding. There is an urgent need to design and implement particular national interventions to prevent and reduce the incidence of PV in schools in Rwanda. PV was also associated with poor parent-child relations. Parents and other adult caregivers should be sensitised to the consequences of PV on children and be urged to adopt positive parenting practices.

## Introduction

Globally, many children are subjected to physical violence (PV) by parents, caregivers, peers, adult relatives or other adults in neighbourhoods [1]. PV is generally defined as the *intentional use of physical force that can lead to death, disability, injury or harm. It encompasses punching, kicking, whipping, beating with an object, choking, suffocating, attempted drowning, intentional burning, using or threatening with a knife, gun or other weapons* [2]. The 2014 global status report on violence prevention estimated that one in four children experienced physical abuse yearly [3]. In some sub-Saharan African countries, surveys on violence against children found higher trends of PV in childhood. In Lesotho, one in three female youth and more than one in two male youth aged 18–24 reported having experienced PV before age eighteen [4]. In Uganda, six in ten female and seven in ten male children reported having experienced PV during childhood [5]. Similar trends were reported in Ivory Coast, with nearly half of females and three in five males aged 18–24 reporting having experienced PV in their childhoods [6].

In Rwanda, until 2015, there were no national data on the prevalence of PV against children. The first national population-based survey on violence against children was conducted in 2015 on children and youth aged 13–24. This survey found that 37% of female youth and 60% of male youth aged 18–24 had experienced PV before age 18. It also found that 27% of females and 42% of males aged 13–17 years had experienced PV in the past 12 months before the survey [7].

As in the other sub-Saharan African countries mentioned above, data from the Rwanda survey showed that PV against children was worrisome and required appropriate interventions and strategies to curb it. According to the World Health Organization (WHO), the design and implementation of any interventions or programs to prevent violence against children have to follow some steps: the problem definition, the identification of protective and risk factors, designing interventions and testing their implementation, the dissemination of information about the effectiveness of interventions, and the scale-up of interventions proven to be effective [8]. With the 2015 survey, Rwanda had just started defining the problem but needed to go further to comply with the WHO-recommended process for violence prevention. To contribute to Rwanda’s efforts towards preventing violence against children, this study used the 2015 Rwanda survey data to assess patterns of PV against children in Rwanda and investigate the associated factors.

## Study objectives

The main objective of this study was to contribute to efforts to fill existing data gaps on the characteristics of PV against children in Rwanda. Additional knowledge about the manifestation of PV against children in Rwanda would inform steps in designing and implementing interventions for its primary prevention. Two specific objectives were also pursued:

- To describe patterns of the prevalence of PV in children in Rwanda
- To investigate factors associated with PV in children in Rwanda.

## Method

As mentioned above, analyses presented in this study are based on data from a survey on violence against children and youth conducted in Rwanda in 2015.

### Survey background

The Rwanda violence against children and youth survey is a nationally representative cross-sectional study done by the Rwanda Ministry of Health in 2015 to produce estimates of the national prevalence of sexual, emotional, and physical violence among female and male children and youth in Rwanda. It adapted and used tools of the violence against children surveys (VACS) that have been developed by the Centers for Disease Control and Prevention (CDC) in the United States of America to support the global efforts for the surveillance of violence against children [9]. VACS aims to help countries generate national-level data that can inform the development and implementation of effective national strategies to prevent violence in children and provide appropriate support to its victims. They produce estimates on childhood prevalence and past-year incidence of physical, emotional and sexual violence among male and female children. They also collect contextual information and data on the occurrences of violence, its risk and protective factors, perpetrators, health and socioeconomic outcomes for victims, as well as their knowledge and use of public health and support services. CDC provides technical support in the design and implementation of VACS across countries to ensure compliance with ethical and safety recommendations of the World Health Organization (WHO) on how to obtain informed consent for participation in surveys that contain questions on domestic violence to protect the safety of both the respondent and the interviewer [10]. The Rwanda survey also benefitted from technical support from CDC and UNICEF-Rwanda.

### Survey participants, selection and sample size

A total sample of 1,180 males and 1,032 females participated in the Rwanda survey. Eligibility criteria included being a male or female aged 13–24 years in a selected household and being able to speak English or Kinyarwanda. Children and youth with mental disabilities, who could not understand and respond to the questions being asked, and those with hearing and speech impairment preventing them from participating in the oral survey interview were excluded from the survey. To compensate for that limitation for children and youth with disabilities, a separate qualitative violence survey was conducted among children and youth with disability in care institutions in Rwanda [11].

To recruit its participants, the Rwanda survey applied a three-stage, split-sample design to obtain female and male samples. The first stage consisted in selecting 250 enumeration areas (EAs) from a list of 14,837 villages in Rwanda, using the probability proportional to size techniques. The 250 selected EAs were stratified by sex, 111 EAs for female participants and 139 EAs for male participants, to account for the split-sample design. The differences were based on varying anticipated response rates by sex and household screening rates [12]. Splitting samples is a technique recommended in VACS to protect respondents’ confidentiality and eliminate the chances that a perpetrator and victim of sexual violence would be interviewed [13]. The second stage of the sampling process consisted of selecting a cluster of 25 households using equal probability systematic sampling in each enumeration area. In the last sampling stage, one eligible respondent, female or male, aged 13–24 years, was selected randomly from all eligible females (or males) in each selected household. The overall individual response rate was 98.30% in female participants. That is to say, 1032 female participants completed the survey of 1050 eligible female participants. It was 98.70 in male participants; that is to say, 1180 male participants completed the survey of 1196 eligible male participants.

VACS participants aged 13–17 years are considered children, and those aged 18–24 are referred to as youth. Analyses presented in this study assessed the category of children only. Thus, we analysed a sample of 1,110 children (618 boys and 492 girls) aged 13–17.

### Survey tools

Rwanda’s survey adapted and applied tools and methods of VACS. The VACS design consists of two questionnaires. The first questionnaire is called the “Household Questionnaire” and is administered to the head of household or any available adult who can respond on behalf of the head of household. It collects data on basic household demographics and assesses whether there are any vulnerable children in the household. The second questionnaire is the “Respondent Questionnaire” and is administered to eligible female or male respondents selected in each sampled household. It covers several topics: demographics, parental relations, family, friends and community support, school experiences, physical violence, emotional violence, and sexual violence [14]. Both questionnaires were adapted and utilised by the Rwanda survey.

### Survey administration

Trained data collectors collected data through face-to-face interviews either in Kinyarwanda or English. Female interviewers interviewed female participants, and male interviewers interviewed male participants. Upon entering a randomly selected household, interviewers identified the head of household and determined the eligibility of household members to participate. The household head was invited to participate in the head of the household interview to assess the household’s socioeconomic conditions. In case more than one eligible participant was available in the selected household, interviewers selected one respondent using a random selection program installed on the netbooks. For households without eligible participants, the head of the household was still asked to participate in the household questionnaire. When the selected respondent was not readily available to participate in the interview, the interviewer revisited the household when the selected respondent would be available. If the selected respondent could not be available after three attempts or refused to participate, the household was skipped regardless of whether another eligible respondent existed; neither the household nor the eligible respondents were replaced [7].

The interviews were conducted in a private setting for confidentiality. During interviews, electronic netbooks with CSPro software were used in data collection to facilitate the management of many skip patterns and logic sequencing in the questionnaire. After field data collection, data were extracted from the netbooks, checked and cleaned for missing or incomplete data and outliers. STATA 13 was used for all data-cleaning processes.

### Ethical considerations

The Rwanda survey adhered to ethical and safety recommendations of the World Health Organization (WHO) on obtaining informed consent for participation in surveys that contain questions on domestic violence to protect the safety of both the respondent and the interviewer. Thus, the survey was not introduced to the household and community as a survey on violence. To avoid reference to any violence happening in the home, the survey was introduced and presented to parents/primary caregivers very broadly, and ‘community violence’ was only mentioned as part of a list of broad topics, such as access to healthcare services and education [9].

Data collectors requested and obtained participants’ consent before survey administration. There was a two-step process to obtain consent. First, the interviewer asked for the head of the household’s consent to conduct the survey in the selected household and to participate in the household questionnaire interview. Secondly, the interviewer requested the consent of child/youth respondents. The interviewer received assent from the minor respondents (13–17 years old) and informed consent from selected respondents who were 8–24 years old. A similar consent process was used in a child-headed household, except that parental/caregiver permission was unnecessary.

Once an eligible respondent was selected in the household, the interviewer read the contents of an initial information form that introduced the survey as an opportunity to learn more about young people’s health, educational and life experiences. After the interviewer and respondent ensured privacy, the interviewer read the contents of a verbal consent form. This informed the respondents that the information they provided was anonymous and that their decision to participate was voluntary. Respondents were also told that if they participated, information about their experiences with physical, emotional and sexual violence would be asked. Respondents were informed that the information they shared was confidential and would not be shared with anyone. They were informed that the only confidentiality exception was if they told the interviewer they were planning to hurt themselves or someone else or if an adult was hurting them. In those cases, the interviewer had to provide them with a mandatory referral to a social worker. This ensured compliance with Rwanda’s mandatory reporting law for child abuse. After reading the information and consent form [7], informed verbal consent was obtained from each respondent.

The survey protocol and data collection tools were independently reviewed and approved by the CDC’s Institutional Review Board and the Rwanda National Ethics Committee (RNEC).

### Data analysis

#### Outcome measure: PV

Self-reported PV was the outcome measure in this study. Four categories of PV were asked about, considering four types of PV potential perpetrators: (a) intimate partners; (b) peers; (c) parents, adult caregivers or other adult relatives; and (d) adults in the neighbourhoods. For each potential perpetrator, three measures of PV were asked about: “Has (i) a romantic partner, boyfriend, or husband; (ii) a person of your age; (iii) a parent, adult caregiver, or another adult relative; (iv) an adult in the neighbourhoods ever: (1) Punched, kicked, whipped or beaten you with an object, (2) Choked, suffocated, tried to drown you, or burned you intentionally? (3) Hurt or threatened you with a knife, gun or other weapons? Respondents who reported having experienced PV were asked about the age at which it happened for the first time and whether it occurred in the last 12 months before the survey 7]. Based on types of PV and the time at which reported PV had happened, an outcome variable called “Physical violence” was constructed for this study. It was defined as having experienced at least one of the four categories of PV assessed by the survey in Rwanda.

#### Independent variables

The following sociodemographic variables were included in our analyses because we considered them perusable, and in addition, previous studies have also shown them to be associated with PV [15]:

- Individual characteristics: Age, gender, Orphan hood status (single or double orphan), schooling status (going or not going to school during the study time), sexually active and romantic relationship.
- Parental relations: living with parents (living with both parents, neither parent, a single parent), closeness with mother (very close, close, not close) closeness with mother (very close, close, not close with), closeness with biological parents (very close, close, not close with).
- Household socioeconomic characteristics: age of the head of household (aged less than 30 years, 31 years and above), gender of the head of household, household size, household wealth index, and household health insurance (has insurance, no insurance).
- Community relationships: Friendship (No friend, has more than one friend), talking to friends (talks to friends a lot, talks to friends somewhat, does not talk to friends), community safety (very safe, somewhat safe, not safe), and community trust (trust much, some trust, no trust).

### Statistical analyses

A total sample of 1,110 children (618 boys and 492 girls) aged 13–17 was analysed. All statistics (descriptive statistics and logistic regression models) were adjusted using standard weighting procedures to correct for unequal probability of selection and change for non-response and to produce national results representative of the national population of children aged 13–17 years in Rwanda. The weighting procedure was applied in two steps. In the first step, a base weight for each sample respondent was performed. In the second step, base weights for non-response were adjusted for [12]. We reported weighted percentages with confidence intervals (CI) by gender in descriptive statistics results. Predictors of PV were investigated using multivariate logistic regression models. A manual backward elimination model selection was conducted to obtain the final reduced logistic regression model, and variables statistically significant at an alpha < 0.1 in the full model were all kept in the final reduced model. Odds ratios (ORs) produced in the full and reduced logistic regression models were considered statistically significant at an alpha < 0.05. Bonferroni correction test was used to adjust *p*-values and the 95% CI for multiple comparisons to ensure that reported *p*-values and CI were not just by chance. Analyses were performed in Stata 14.2.

## Results

### Descriptive characteristics

Over three in ten male children (36.53%) and more than two in ten female children (23.38%) reported having experienced any form of PV in their lifetime. In the same way, slightly over two in ten male children (21.81%) and slightly over one in ten female children reported PV within the last twelve months before the survey. Of male children who reported PV in the previous 12 months before the survey, 60% (59.99%) had experienced multiple events of PV. More details about the prevalence of PV in children in Rwanda are presented in Table 1.

**Table 1:** PV reported by 13–17-Year-Old Children, by Gender: Rwanda Violence Against Children and Youth Survey, 2015

**Forms of PV**	**Recent PV** **(Last 12 months)**		**Lifetime PV** **(Ever physically abused)**	
	**Male children (** ***N*** ** = 618)**	**Female Children (** ***N*** ** = 492)**	**Male children (*N* = 618)**	**Female Children (*N* = 492)**
	**%**^**a**^	**%**^**a**^	**%**^**a**^	**%**^**a**^
**Reported any PV**	21.81	12.73	36.53	23.38
**Physical violence by peers**	8.71	5.39	21.36	12.68
**Physical violence by a parent, adult caregivers or other adult relatives**	11.36	5.75	28.34	15.77
**Physical violence by adults in the community**	12.67	3.46	20.15	6.69
**Physical violence by intimate partner among those with an intimate partner (Male: *n* = 164, Female: *n* = 107)**	2.33	2.09	4.905	3.374
**Multiple events of PV among those who experienced at least one occurrence of Physical Violence (Male: *n* = 259, Female: *n* = 137)**	59.99	32.02	54.68	33.31

^a^ Nationally representative weighted percentage

### Background characteristics of children aged 13–17 years who reported any PV in the last 12 months before the survey

The majority of children who reported PV in the last twelve months before the survey were aged 13 years: 22.97% in male children and 15.57% in female children. Approximately 40% of children reported PV (23.41% in male children and 17.13% in female children) did not feel close to both biological parents. More details about the distribution of physically abused children’s background characteristics are presented in Table 2.

**Table 2:** Background characteristics of children aged 13–17 who reported any PV in the last 12 months by gender: Rwanda Violence Against Children and Youth Survey, 2015

**Background characteristics**	**Male Children**	**Female Children**
	**(*n*** **= 258)**^a^	**(*n*** **= 137)**^a^
	**%** ^b^	**%** ^b^
**Child age**		
**Younger (13–15 Years)**	34.62	21.31
**Older (16–17 Years)**	28.53	15.53
**Education**		
**Attends schools**	54.04	28.79
**Does not attend school**	9.04	8.13
**Orphan**		
**Not an orphan**	50.38	30.62
**Orphaned**	12.59	6.40
**Has a romantic partner**		
**No**	45.60	30.41
**Yes**	18.32	5.67
**Sexually active**		
**No**	54.23	33.77
**Yes**	8.92	3.08
** Friendship**		
**Has friends**	59.46	33.24
**No friends**	3.69	3.602
**Talking to friends**		
**Talks to friends a lot**	18.35	12.24
**Talks to friends somewhat**	31.38	15.26
**Does not talk to friends**	13.43	9.35
**Trusting people in the community**		
**Trust people a lot**	25.27	11.01
**Trust people somewhat**	21.79	12.19
**Does not trust people**	16.09	13.65
**Safety in the community**		
**Feels very safe**	34.33	15.14
**Feels somewhat safe**	26.94	17.70
**Does not feel safe**	1.88	4.00
**Living arrangement**		
**Live with both parents**	35.86	23.29
**Live with neither parent**	10.13	4.95
**Live with a single parent**	17.23	8.54
**Closeness with father**		
**Very close with father**	24.77	10.77
**Close with father**	17.17	10.37
**Not close with father**	21.21	15.70
**Closeness with mother**		
**Very close with mother**	45.03	22.27
**Close with mother**	11.91	7.93
**Not close with mother**	6.22	6.64
**Closeness with both parents**		
**Very close with both parents**	20.13	8.53
**Close with both parents**	19.62	11.19
**Not close with both parents**	23.41	17.13
**Gender of the head of household**		
**Male**	45.26	28.29
**Female**	17.90	8.56
**Age of the head of household**		
**< 30 years**	2.58	2.35
**31 years and more**	60.58	34.50
**Household size**		
**1–4 People**	15.84	7.51
**Five people and more**	47.32	29.34
**Household wealth**		
**Higher wealth quintile**	23.42	6.77
**Middle wealth quintile**	21.93	15.64
**Lower wealth quintile**	18.15	14.09
**Households covered by a health insurance**		
**Yes**	47.52	27.19
**No**	15.63	9.65

^a^ Number of male and female children who reported any PV in the last 12 months before the survey

^b^ Nationally representative weighted percentages

### Predictors of PV against children in Rwanda

Table 3 presents odds ratios (OR) of factors assessed for associations with PV against children in Rwanda. Odds ratios of the reduced model indicated that older age (OR: 0.53, 95% CI [0.40–0.72]), being a female child (OR: 0.43, 95% CI [0.31–0.58]), not attending school (OR: 0.48, 95% CI [0.31–0.73]), living in a household head by a person older than 30 years (OR: 0.48, 95% CI [0.25–0.92]), and living in a female-headed household (OR: 0.58, 95% CI [0.38–0.89]) were negatively associated with PV against children.

**Table 3:** Predictors of PV among all 13–17-year-old Children, Rwanda Violence Against Children and Youth Survey, 2015

**Predictors**	**Full Model**			**Reduced Model**		
	**OR**	**[95%CI]**	***P*** **-Value**	**OR**	**[95%CI]**	***P*** **-Value**
**Child gender**						
**Male**	Ref.			Ref.		
**Female**	**0.43**	**[0.31–0.58]**	0.001*	**0.43**	**[0.31–0.58]**	0.001*
**Child age**						
**Younger (13–15 Years)**	Ref.			Ref.		
**Older (16–17 Years)**	**0.53**	**[0.39–0.72]**	0.001*	**0.53**	**[0.40–0.72]**	0.001*
**Schooling status**						
**Attends school**	Ref.			Ref.		
**Does not attend school**	**0.50**	**[0.33–0.78]**	0.001*	**0.48**	**[0.31–0.73]**	0.001*
**Sexually active**						
**No**	Ref.			Ref.		
**Yes**	**1.68**	**[1.05–2.69]**	0.03*	**1.66**	**[1.05–2.63]**	0.03*
**Number of friends**						
**No friends**	Ref.			Ref.		
**One friend and more**	0.97	[0.57–1.64]	0.89			
**Talking to friends**^ß^						
**Talks to friends a lot**	Ref.					
**Talks to friends somewhat**	0.79	[0.53–1.18]	0.48			
**Does not talk to friends**	0.72	[0.45–1.16]	0.31			
**Trusting people in the community**^ß^						
**Trust people a lot**	Ref.			Ref.		
**Trust people somewhat**	0.7	[0.42–1.17]	0.29	0.62	[0.39–0.98]	0.04*
**Does not trust people**	1.08	[0.60–1.95]	1.00	0.96	[0.60–1.55]	1.00
**Safety in the community**^ß^						
**Feels very safe**	Ref.					
**Feels somewhat safe**	0.85	[0.54–1.32]	1.00			
**Does not feel safe**	1.23	[0.51–2.98]	1.00			
**Living arrangement**^ß^						
**Live with both parents.**	Ref.			Ref.		
**Live with neither parent**	1.27	[0.69–2.34]	1.00	1.3	[0.73–2.32]	0.82
**Live with a single parent**	1.66	[0.94–2.91]	0.1	1.65	[0.95–2.87]	0.09
**Closeness with both parents**^ß^						
**Very close with both parents**	Ref.			Ref.		
**Close with both parents**	2.11	[0.85–5.23]	0.14	1.77	[1.15–2.71]	0.01*
**Not close with both parents**	2.00	[0.54–7.38]	0.6	2.14	[1.31–3.49]	0.001*
**Closeness with father**^ß^						
**Very close with father**	Ref.					
**Close with father**	0.85	[0.38–1.91]	1.00			
**Not close with father**	1.13	[0.34–3.77]	1.00			
**Closeness with mother**						
**Very close with mother**						
**Close with mother**	0.95	[0.54–1.66]	1.00			
**Not close with mother**	1.02	[0.47–2.20]	1.00			
**Gender of the head of household**						
**Male**	Ref.			Ref.		
**Female**	**0.58**	**[0.38–0.88]**	0.01*	**0.58**	**[0.38–0.89]**	0.01*
**Age of the head of household**						
**< 30 years**	Ref.			Ref.		
**31 years and more**	**0.48**	**[0.25–0.91]**	0.03*	**0.48**	**[0.25–0.92]**	0.03*
**Household size**						
**1–4 People**	Ref.			Ref.		
**Five people and more**	**1.53**	**[1.04–2.23]**	0.03*	**1.55**	**[1.07–2.26]**	0.02*
**Household wealth index**^ß^						
**Higher wealth quintile**	Ref.			Ref.		
**Middle wealth quintile**	**1.62**	**[1.06–2.46]**	0.02*	**1.63**	**[1.08–2.47]**	0.02*
**Lower wealth quintile**	1.35	[0.83–2.20]	0.42	1.35	[0.84–2.15]	0.38
**Covered by a health insurance**						
**Yes**						
**No**	0.97	[0.68–1.38]	0.88			

Observations: 1,092

*OR* Adjusted odds ratios, *CI* Confidence Intervals

^ß^ Reported *p*-values and CI have been adjusted for multiple comparisons with Bonferroni correction Test

* Estimated Odds Ratios highlighted in bold are statistically significant at *P*-value < 0.05

On the other hand, the odds of reporting PV increased with being sexually active (OR: 1.66, 95% CI [1.05–2.63]), being in a household from the middle wealth quintile (OR: 1.63, 95% CI [1.08–2.47]); not feeling close to both biological parents (OR: 2.14, 95% CI [1.31–3.49]), and living in a larger household of more than five people (OR: 1.55, 95% CI [1.07–2.26]). Additional details on predictors of PV against children in Rwanda are presented in Table 3.

## Discussion

This study pursued two specific objectives: to describe patterns of the prevalence of PV against children in Rwanda and investigate associated factors. Regarding the first objective, our findings indicated that PV against children was widespread in Rwanda. Significant differences were observed in the occurrence of PV in male children and female children. Except for PV by an intimate partner, whose prevalence was approximately the same among male and female children, the reported prevalence for each of the other forms of PV considered by this study was significantly higher in male children than in female children. In addition, we found that younger children were more likely to be physically abused than older children. These two findings on younger child age and gender differences in the occurrence of PV against children in Rwanda were expected and are consistent with results from similar studies. Several studies have documented evidence that younger and male children experience more PV than older children and female children [16–19].

We also found that children in school were six times more likely to report PV than children out of school. The higher prevalence of PV observed in schooling children than in children out of school might partly be explained by the effect of group dynamics. Unlike children out of school, children attending school belong to social groups composed of people from different backgrounds who need to interact with and influence one another. The integration and interaction process in a group is often tested by the conflict between group members [20]. In that context, as with other human social groups, the school environment has an inherent potential for generating conflict, frustration and violent responses among its members [21]. UNESCO has alluded to that reality and defined school violence as “*all forms of violence that takes place in and around schools and is experienced by students and perpetrated by other students, teachers and other school staff*” as school violence [22].

Another interpretation of this finding on the prevalence of PV in schooling and non-schooling children in Rwanda would be the persistence and tolerance of corporal punishment as one way to discipline children at school and home. A study conducted on the perception of parents and teachers on the practice of corporal punishment in primary schools in Rwanda in 2019 found that many parents and teachers believed that corporal punishment was a good way to punish, discipline and educate children [23]. In addition, while corporal punishment is prohibited by law, there is evidence that it is tolerated at schools and at home as a way to discipline children [24].

As for the second objective of this study, we investigated factors associated with PV against children in Rwanda. We found that children who did not feel close to both biological parents, children from the middle wealth quintile and children from larger households of more than five people had greater odds of being physically abused than their fellow who felt very close to their biological parents, children from the higher wealth quintile, and children from smaller households. These findings corroborated facts from other studies that showed that poor relationships between children and biological parents might lead children to social isolation and expose them to victimisation [13]. In most cases, the lack of a close relationship between children and biological parents can result from poor parenting practices or other family stressors [11]. There is still a debate on the effect of larger households on the risk for PV [12], but some other studies have revealed that larger households can increase the risk of PV against children [25]. Regarding household wealth, studies conducted in Nigeria and Uganda found that children from poor and socioeconomically disadvantaged households had an increased risk of being victims of PV [26, 27]. Other studies have demonstrated that children from households with disadvantaged socioeconomic backgrounds tend to experience stress, depression, and conflict in their relationships, all of which compromise their behaviours and increase the risk for violence and adverse childhood experiences [28].

Last, this study found that children in households headed by females were less likely to report PV than children from male-headed families. This finding corroborates the effects of gender roles in the perpetration of PV against children [8]. It could indicate the persistence of patriarchal and masculine norms regarding parenting practices in Rwandan society[29–32]. According to Rwanda’s traditional and cultural norms, fathers are de facto heads of household and custodians of order in the home, including disciplining children for misbehaviour [33].

### Limitations

This study has several limitations related to its design, which should be considered when interpreting its findings. First, the data analysed in this study are self-reported. Some biased responses might have been provided due to the misunderstanding of what was asked or desirability bias for respondents who would have wanted to look good [34]. Second, being a cross-sectional study, it was impossible to determine direct causal relationships of social relations studied with PV.

## Conclusion

The findings in this study indicated that PV is widespread in Rwanda, especially in school settings. There is a need to design and implement particular national interventions to prevent and reduce the incidence of PV in schools in Rwanda. Factors associated with PV against children in Rwanda include individual characteristics, parent-child relational factors and socioeconomic factors. To reverse the incidence of PV against children in Rwanda, relevant institutions need to organise awareness-raising campaigns to denounce PV and its consequences on children and promote positive parenting attitudes for parents and caregivers.

This study did not assess specific contexts leading to poor close relationships between children and parents. However, future studies on child maltreatment in Rwanda can explore that aspect for more insights into that issue. We also recommend further investigating drivers of higher rates of physical violence in schools and proposing remedial actions.

## Data Availability

The datasets used and analyses performed for this study are available from the corresponding author (Alypio Nyandwi; Email: nalypio@gmail.com) and can be obtained on reasonable request.

## Abbreviations

PV: Physical Violence
VACS: Violence Against Children Surveys
CDC: The Centers for Disease Control and Prevention
WHO: World Health Organization
EAs: Enumeration Areas
RNEC: Rwanda National Ethics Committee

## Declarations

The survey protocol and data collection tools used by the Rwanda violence against children and youth survey were independently reviewed and approved by the CDC’s Institutional Review Board and the Rwanda National Ethics Committee (RNEC).

The survey adhered to all procedures recommended and applied in studies involving human participants by the ethical standards of the institutional and national research committee and with the 1964 Helsinki declaration and its later amendments or comparable ethical standards.

Verbal voluntary informed consent was obtained from all participants included in the survey. Verbal informed consent to participate in the survey was obtained from participants aged 18–24, and parents provided consent for participants aged 13–17. The latter also offered their verbal assent. A two-step process was followed to obtain the consent of study participants. First, data collectors requested permission from the head of the household to survey the selected household. After the approval of the head of the household, the data collector interviewed him with the head of the household questionnaire. After that, the data collector proceeded with requesting and obtaining the consent of child/youth respondents. The interviewer received informed assent from minors (13–17 years old). The same informed consent process was used in emancipated respondents (18–24 years old) and those who lived in a child-headed household, except parental/caregiver permission was unnecessary. Verbal voluntary consent was preferred because data collection was done with electronic devices.

It was explained to the survey participants that the information they provided was anonymous, that their participation was voluntary, and that they could opt out of the involvement in survey data collection.
